# Familiarity, Attitude, and Confidence of Health Literacy Practice among Community Healthcare Providers in Taiwan

**DOI:** 10.3390/ijerph182312610

**Published:** 2021-11-30

**Authors:** Mei-Chuan Chang, Jyh-Gang Hsieh, Mi-Hsiu Wei, Chuan-Hsiu Tsai, Jui-Hung Yu, Ying-Wei Wang

**Affiliations:** 1 Department of Nursing, Tzu Chi University, Hualien 970, Taiwan; meichuan2@gmail.com (M.-C.C.); janechtsai@gmail.com (C.-H.T.); 2 Department of Family Medicine, Hualien Tzu Chi Hospital, Buddhist Tzu Chi Medical Foundation, Hualien 970, Taiwan; jyhgang@gmail.com; 3 Department of Medical Humanities, School of Medicine, Tzu Chi University, Hualien 970, Taiwan; 4 Department of Communication Studies, Tzu Chi University, Hualien 970, Taiwan; michelle@mail.tcu.edu.tw; 5 Department of Public Health, Tzu Chi University, Hualien 970, Taiwan; jhy@mail.tcu.edu.tw

**Keywords:** community healthcare providers, health literacy, familiarity, attitude, confidence

## Abstract

Health literacy (HL), which is a determinant of individuals’ health as well as a personal and public asset, can be improved by community healthcare providers (CHPs) with the capability of providing HL services. The purpose of this study was to explore CHPs’ familiarity with and attitudes toward HL and their confidence in implementing HL practices. A cross-sectional online survey was conducted involving a total of 104 CHPs from 20 public health centers in Taiwan. It was based on a structured questionnaire involving self-evaluation by participants. The scores for familiarity, attitudes, and confidence in implementing HL practices were mean = 4.36, SD = 1.99; mean = 7.45, SD = 1.93; and mean = 6.10, SD = 1.77 (out of 10 points), respectively. The results of the multiple regression analysis showed that the two independent variables of familiarity and attitude could predict confidence in implementing HL practices (R^2^ = 0.57, F(2101) = 58.96, *p* < 0.001). The CHPs surveyed were not especially familiar with HL; thus, they recognized its importance, but they lacked confidence in implementing HL practices. Increasing practitioners’ familiarity with HL may therefore boost their confidence in implementation. The research results can serve as a reference when planning HL education and training.

## 1. Introduction

Health literacy (HL) is composed of “the personal skills that enable individuals to obtain, understand, and use information to make decisions and take actions that will have an impact on their health”. It is an important determinant of individuals’ health and can be regarded as a personal and public asset from the healthcare promotion perspective. It can be cultivated through educational, organizational, and other interventional measures, and its ability to predict health surpasses that of the other factors affecting health [1]. The cultivation of HL does not only involve individuals’ efforts to enhance their abilities but also those of healthcare organizations and personnel, who can assist the public in finding, understanding, and using information and services to achieve HL [2,3]. Therefore, HL professionals have become important agents of the implementation strategies of various countries’ HL action plans.

Many researchers have stated that the professional competencies of healthcare personnel in HL extend beyond a mere understanding of the topic to include HL skills, such as the provision of clear and easily comprehensible information, application of teach-back practices, utilization of comprehensive HL prevention methods, maximization of patient participation in healthcare, incorporation of HL into the collaboration model for cross-professional teams, and improvement of healthcare environments to render them shame-free [4,5,6,7]. Although the importance of improving healthcare services in terms of HL enhancement has been emphasized for many years, many recent studies have showed that clinical medical personnel from various countries still lack an in-depth understanding of HL [4,7]. The personnel studied did not have an adequate understanding of the HL concept, were unclear of the ways in which to apply HL strategies and evaluate patients with low levels of HL, lacked knowledge of the applicable written teaching materials, and held negative attitudes toward the practice of HL-related care [8,9,10,11,12]. Moreover, HL has rarely been a topic of discussion in the healthcare context [7].

Efforts to enhance the public’s HL are not limited to medical and healthcare organizations; rather, more importantly, they should begin in the communities where people live. In 2019, the European Office of the World Health Organization issued a draft strategy for implementing HL based on the life cycle and pointed out that health-literate communities and cities provide people with trustworthy healthcare information. Thus, healthcare choices are made easier, which positively affects and promotes enhanced health and healthy living [13]. The German HL Action Plan pointed out that enhancements to HL and improvements to the HL environment should be carried out based on consideration of people’s daily lives [14].

Community healthcare providers (CHPs), such as community health nurses, physicians, dietitians, and health administrators, are critical implementers of community healthcare promotion and HL enhancement [15]. Unlike professionals in medical organizations, CHPs serve both individuals and groups, and they have the important task of transmitting healthcare information [16]. According to the literature, the capabilities emphasized in the promotion of HL practices by CHPs include understanding the ways in which service recipients acquire information, providing multiple channels for the transmission of information, appropriately communicating verbal and written information, and emphasizing the population’s cultural sensitivities and public participation [17,18]. Therefore, understanding CHPs’ knowledge of and attitudes toward health literacy as well as their ability to implement health literacy-related practices is of vital importance to improving the health literacy of communities.

In Taiwan, approximately 30–50% of the population has insufficient or limited HL [19,20]. The CHPs at public health centers are the frontline staff dealing with the provision of primary care and promotion of healthy living in communities. They provide the public with information on various important issues, including health promotion campaigns, health education activities, and disease prevention measures [15]. These tasks involve the transmission of a substantial amount of health-related information and services. An important aspect of their ability to improve service quality involves considering the issues that people have with HL. Most of the existing literature has been focused on the practical abilities of the clinical medical personnel who work in medical organizations, but there is a relative lack of research on CHPs’ practical abilities regarding HL. 

HL is a new concept for service providers in many countries’ healthcare systems. Community healthcare emphasizes the health of both individuals and groups and therefore requires a different information transmission process from that used in the hospital-based communication model. The first step in planning programs for the training of CHPs in HL education is to gain a deeper understanding of their current level of knowledge of the topic and abilities. The main purpose of this study was therefore to understand the confidence level of CHPs in implementing HL practices and the related factors affecting it. The findings can serve as a reference when planning HL education and training for CHPs.

## 2. Materials and Methods

The research design of this study was a cross-sectional survey.

### 2.1. Participants

The participants of this study were enrolled in the health literacy education and training program held by the Health Promotion Administration in 2020. The 20 public health centers volunteered to take part in the course and were selected from northern, central, southern, and eastern Taiwan as well as the offshore islands according to Taiwan’s geographical distribution. Approximately 100–150 CHPs at these public health centers were invited to take part in a survey, conducted via an anonymous electronic questionnaire. The inclusion criteria of the participants were that they needed to be 20 years of age or older and their workplace needed to be a community public health center or healthcare services center. The exclusion conditions were being a temporary worker or intern at those centers. A total of 107 responses were received, including 104 valid ones.

### 2.2. Research Tools

To develop the measurement tool for this study, the researchers referred to the relevant literature and considered the attributes of community healthcare work in Taiwan, including demographic variables and the subjects’ familiarity with, attitudes toward, and confidence in implementing HL practices. Once the draft questionnaire had been prepared, five experts in related fields, including health communication, community healthcare, and HL, were invited to conduct the content validity testing. The main aim was to evaluate the applicability and clarity of the questionnaire items and calculate the experts’ content validity index (CVI). The CVI values for applicability and clarity were 0.97–1.0 and 1.0, respectively. The reliability of the tool was also tested for internal consistency, with the Cronbach’s alpha value being 0.97–0.98. The finalized version of the questionnaire contained 9 items on demographic variables, 10 items on familiarity with HL, 5 items on attitudes toward HL, and 8 items on confidence in implementing HL practices (with an additional 8 and 4 items on verbal and written communication under its sub-scales, respectively). Each item was measured using an 11-point scale (except the items for demographic variables), with 10 points indicating “very familiar/agree/confident” and 0 indicating “very unfamiliar/disagree/not confident”. The scores for the values pertaining to each item were based on the participants’ self-evaluations.

### 2.3. Data Collection and Analysis

The online questionnaire was administered in Chinese using Google Forms, and anonymity was maintained. A QR code was prepared based on the questionnaire’s web page and sent to the participants. Duly responding to all the items was required before the questionnaire could be submitted. After completion of the data collection process, the Excel data file was converted to SPSS 19.0 for the data analysis. The analytical methods included descriptive statistics, an independent sample *t*-test, a single-factor analysis of variance (ANOVA), and multiple regression analyses. The frequencies and percentages of demographic variables were presented by descriptive statistics; the score of each measurement question item was expressed as a mean and standard deviation. The main variables, such as the scores for familiarity with health literacy, attitudes toward it, and self-confidence in the implementation of related practices, were expressed as a mean and standard deviation derived from combining the scores of all the question items that measured each variable and then dividing the score by the number of question items; for inter-group comparisons of demographic variables, the *t*-test and ANOVA methods were applied for continuous variables; a correlation analysis of two continuous variables was conducted using a Pearson product-moment correlation. Multiple regressions were used to analyze the relationship between the various variables and the CHPs’ confidence in implementing HL practices. The residual value of the normal probability plot, Kolmogorov–Smirnov test, Durbin–Watson test, Levene’s test, and variance inflation factor (VIF) were used to test the assumptions of linear regression.

### 2.4. Research Ethics

This study was reviewed by the medical institution’s research ethics committee. The IRB license number is IRB109-151-B.

## 3. Results

### 3.1. Demographic Variables

The demographic variables of the participants are shown in Table 1. Most (95) were female, accounting for 91.3% of the total sample. Most (75.0%) were between 31 and 50 years old, with the average age being 41.1, SD = 9.53. In terms of educational qualifications, 79.8% (83) possessed a college degree or above. In terms of job title and description, 73.1% (76) were nurses and 12.5% (13) had supervisory duties. The average length of work experience was 15.19, SD = 9.05 years, with 11–20 years being the predominant tenure, at 43.3% (45). Among the participants, 40.4% (42) had not attended any previous HL-related courses, while 51.9% (54) had attended HL courses of fewer than 10 h in duration.

### 3.2. CHPs’ Familiarity with, Attitudes toward, and Confidence in Implementing HL Practices

The CHPs’ familiarity with HL, shown in Table 2, was not generally high (mean = 4.36, SD = 1.99 out of 10 points). They were the least knowledgeable about the prevalence of people with insufficient HL in the population (mean = 3.97, SD = 1.92). In general, their attitude toward HL practices tended to be positive (mean = 7.45, SD = 1.93). The mean score for confidence in implementing HL practices was 6.10, SD = 1.77. The items with the lowest and highest confidence scores were “able to apply the relevant behavioral theories when designing information content (for transmission to the subjects to positively influence their health behaviors)” and “able to apply the appropriate verbal communication skills when delivering health-related information to the public”, respectively. The average overall score for the verbal communication skills subscale was 6.51, SD = 1.85. The lowest and highest scores were for the items “able to confirm service recipients’ understanding of teach-back” and “(when necessary) communication subjects should encompass the family members or, at least, the main caregiver”, respectively. The average overall score for the written communication subscale was 5.95, SD = 1.87. The item with the lowest score was “able to develop or produce health education materials that are appropriate for the service recipients”; the item with the highest score was “able to help the public locate or select written pamphlets containing HL-friendly materials or service information”.

### 3.3. Inter-Group Differences and Correlations for the Various HL Variables

Single-factor ANOVA and *t*-test analyses were used to compare the various variables, including the participants’ educational level, whether they held supervisory duties, the region where they practiced, and HL education and training as well as inter-group differences in terms of their familiarity with, attitudes toward, and confidence in implementing HL practices. A Pearson’s correlation analysis was also carried out on the relationship between the participants’ responses and their age and length of work experience. The results are shown in Table 3.

There was no difference in or correlation between the participants’ demographics (including educational level, presence of supervisory duties, region where they worked, age, and length of work experience) and their familiarity with, attitudes toward, and confidence in implementing HL practices. The only item in which a difference was evident in the scores for familiarity with HL was HL education and training (*t* = −3.29, *p* < 0.01). The average value indicated that CHPs who had previously participated in HL education and training had a higher degree of familiarity with it than those who had not. 

### 3.4. Factors Affecting the CHPs’ Confidence in Implementing HL Practices

The testing of the assumptions of the linear regression showed that the dependent variable of confidence presented a 45-degree linear distribution in the residual area of the normal probability plot. The results of the Kolmogorov–Smirnov test were not significant and obeyed the assumption of normality. The DW value of the Durbin–Watson test was 2.36, which was greater than the DU value (k = 2, DU_100_ = 1.715), indicating that it obeyed the assumption of independence. The Levene’s test revealed that none of the various independent and dependent variables reached a significant level, which was in line with the homogeneity of variance assumption. The variance inflation factor (VIF) was 1.013 and did not exceed 10, indicating that the probability of finding collinearity among the variables was low. The standardized regression equation indicated that confidence = 0.51 × familiarity + 0.50 × attitude. This model (Table 4) could therefore be used for making good predictions. R^2^ = 0.57, indicating that the two independent variables of familiarity and attitude could explain 57% of the variance in confidence. Familiarity and attitude could predict the CHPs’ confidence level in implementing HL practices F(2,101) = 58.96, *p* < 0.001; the regression coefficient was positive, meaning that the more positive the familiarity and attitude values were, the greater the participants’ confidence levels were.

## 4. Discussion

The results of this study were like those of the surveys conducted by researchers in various countries on the professional HL functions of medical personnel [9,10,11,12]. In summary, CHPs were generally unfamiliar with HL but had an attitude that reflected recognition of the importance of HL practices. The concept of HL first appeared in the related literature in the 1970s [21] but was not discussed in Taiwan until the beginning of 2000 and was not emphasized until 2005 [22]. There are still many disparities among the various equivalent Chinese terms for HL. Literature related to the development and surveys of the measurement tools for Taiwanese HL was not published until after 2010 [23], meaning that HL is still a relatively new topic in Taiwan. In this study, 40% of the participants in the sample had not attended any HL-related courses. Those who had participated in related courses scored higher in terms of familiarity than those who had not. Therefore, it can be concluded that there is a strong demand for HL education and training among CHPs at this stage.

CHPs differ from the medical staff serving in medical institutions: their services are directed toward enhancing the health of the community as a unit in addition to promoting the welfare of the individuals within the community. Correspondingly, their focus and approach to integrating HL into healthcare measures are somewhat different from those of medical organizations. Baur et al. defined community HL interventions as “any purposeful, organized activity to help a group of people find, understand, use, or communicate about health information, services, or issues for themselves or their communities” [16]. Therefore, when confidence in implementing HL practices was measured in this study, the emphasis was placed not only on the individuals’ communication skills, but also on the CHPs’ ability to encourage the participation of individuals and organizations within the community and effectively disseminate public information. Although nearly 70% of the CHPs in this study had more than 10 years of work experience, their average level of confidence in implementing HL practices was only 6.10, SD = 1.77 (out of a total of 10 points), which is not exceptional. 

Among the individual items, the average confidence score for the following was even lower than 6 points: “able to deliver the appropriate written information on health and services”, “able to collect the opinions of service recipients when developing plans or educational materials”, and “able to apply the relevant behavioral theories when designing information content”. Written health education materials are important tools in healthcare education. The channels for disseminating health-related information included leaflets, pamphlets, and online information and were important sources enabling the people in the community to obtain healthcare information. However, many studies have showed that the language used in written health education materials is often difficult for laypeople to understand, the information does not meet their needs, the layout does not facilitate reading comprehension, and the reading level of the information is too high for the average person. This creates barriers to people with insufficient HL obtaining information [24,25]. Many indicators for reviewing written health education materials have been developed to ensure that such information is clear and easily comprehensible [26,27], and they serve as the standards for developing and selecting healthcare information.

In Taiwan, Chang et al. developed the Chinese version of the evaluation indicators and guidelines for health education materials with an emphasis on a user-centric design. This was the first tool in Taiwan to systematically evaluate health education materials from the HL perspective and formed the basis of the standard issued by the Health Promotion Administration for the review of health educational materials [28]. However, the results of this study revealed that CHPs’ average level of familiarity with indicators of written health education materials and their confidence in delivering the appropriate written information on healthcare and the related services were only 4.21 and 5.95 points, respectively (both out of a total of 10 points). There is a need to further promote indicators and principles for composing written health education materials that comply with the requirement of HL-friendliness. This will increase healthcare professionals’ level of awareness, thereby allowing them to provide written information that is applicable to their service recipients.

Many related studies on medical personnel’s HL abilities from various countries have highlighted their lack of understanding of the topic, their inadequate abilities in terms of its practical implementation, and the need for HL education and training, e.g., in the United States [8,9], Turkey [10], Iran [11], and Malaysia [12]. The results of this study confirmed that CHPs who had received education and training possessed a better understanding of HL than those who had not. The analysis also indicated that familiarity with, and attitudes regarding, HL predicted confidence in implementing HL practices. With the transformation in the population’s health problems in recent years, many scholars have revived the discussion of CHPs’ professional competencies and core educational connotations. The dissemination of health-related information, implementation of effective communication, and evaluation of HL have already been proposed [15]. Nevertheless, there is still a need for further planning and discussion of HL education related to community healthcare services, whether through nurturance or on-the-job education.

Although the samples of this study covered the northern, central, southern, and eastern parts of Taiwan as well as the offshore islands, as purposive sampling was adopted, the samples were not adequately representative for generalization; thus, the inferences that can be made based on the results are relatively constrained, which is a limitation of this study.

## 5. Conclusions

Approximately 30–50% of the Taiwanese population has insufficient or limited HL [19,20]. To resolve the HL problem, CHPs play the important role of being frontline providers of healthcare information. This study was focused on using CHPs as research subjects to investigate their level of familiarity with and attitudes toward HL practices as well as their level of confidence in their ability to implement HL services. The results indicated that the health professionals studied were generally unfamiliar with HL. Although they recognized the importance of HL-friendly practices, they lacked sufficient confidence in implementing them. The lowest confidence scores were for developing written information on HL principles, promoting users’ participation, and applying theories to develop information content. Familiarity with and attitudes toward HL were predictors of their level of confidence in implementing HL practices, thereby indicating that there is a need among CHPs for HL education and training. This preliminary study of CHPs’ HL abilities led to the proposal of some initial suggestions regarding their HL-related education and training. Future studies can test and revise the related abilities and examine the impact of education and training on their confidence levels. This will enable CHPs to better perform their role and realize their function of improving the HL of the people in their communities.

## Figures and Tables

**Table 1:** Demographic variables.

Item	Number	Percentage (%)
Gender		
Male	9	8.7
Female	95	91.3
Age		
≤20	6	5.8
21–30	6	5.8
31–40	35	33.7
41–50	43	41.3
≥51	14	13.4
Educational qualifications		
College for professional training	21	20.2
College	72	69.2
Research institute (master’s degree)	11	10.6
Job title		
Physician	1	1.0
Nurse	76	73.1
Medical staff *	4	3.9
Health administrative staff	13	12.5
Other	10	9.6
Presence of supervisory duties		
Yes	13	12.5
No	91	87.5
Length of work experience		
≤10	32	30.7
11–20	45	43.3
≥21	27	26.0
Amount of HL training experience		
None	42	40.4
≤10 h	54	51.9
11–30 h	6	5.8
≥51 h	2	1.9

Note: * Medical personnel include pharmacists, nutritionists, and medical technologists.

**Table 2:** CHPs’ familiarity with, attitudes toward, and confidence in implementing HL practices.

Item		Mean ± Standard Deviation
Familiarity with HL		4.36 ± 1.99
1.	Definition	4.79 ± 1.88
2.	Measurement tools	4.19 ± 2.11
3.	Prevalence of people with insufficient HL (Taiwan)	3.97 ± 1.92
4.	Indicators for identifying people with insufficient HL	4.13 ± 1.92
5.	Impact on health outcomes	4.55 ± 2.09
6.	HL-related communication skills	4.43 ± 1.99
7.	Indicators for HL-friendly health education materials	4.21 ± 2.00
8.	Healthcare literacy environment	4.41 ± 1.99
9.	Universal precautions-based approach to HL	4.53 ± 1.99
10.	Principles of HL and healthcare practices	4.34 ± 2.02
Attitudes toward HL		7.45 ± 1.93
1.	Recognize that HL affects healthcare quality and health outcomes	7.66 ± 1.95
2.	Recognize the importance of identifying people with insufficient HL	7.46 ± 1.87
3.	Recognize the approach based on universal precautions	7.27 ± 1.93
4.	Recognize the necessity of HL in professional competence	7.42 ± 1.98
5.	Recognize the necessity of HL training	7.42 ± 1.93
Confidence in implementing HL practices		6.10 ± 1.77
1.	Able to identify the HL of service recipients	6.15 ± 1.53
2.	Able to assess the ability of service recipients to use social media	6.12 ± 1.69
3.	Verbal communication skills:	6.51 ± 1.85
3.1	Able to use language that is simple and easily understandable	6.63 ± 1.83
3.2	Able to apply chunk and check techniques	6.36 ± 1.77
3.3	Able to confirm service recipients’ understanding of teach-back	6.12 ± 1.82
3.4	Able to apply instructional aids	6.29 ± 1.90
3.5	Able to encourage the asking of questions	6.55 ± 1.74
3.6	Able to prioritize delivery of the most important information	6.63 ± 1.81
3.7	Able to ensure that, when necessary, the communication subjects should encompass the family members or, at least, the main caregiver	6.83 ± 1.95
3.8	Able to actively seek translation assistance when facing language difficulties	6.63 ± 2.01
4.	Written communication skills:	5.95 ± 1.87
4.1	Able to state the standards that written health education materials must meet	6.03 ± 1.94
4.2	Able to help the public locate or select written pamphlets containing HL-friendly materials or service information	6.17 ± 1.83
4.3	Able to judge the suitability of health education materials	5.96 ± 1.79
4.4	Able to develop or produce health education materials that are appropriate for the service recipients	5.65 ± 1.89
5.	Able to collect the opinions of service recipients when developing plans or educational materials	5.87 ± 1.85
6.	Able to apply multiple methods when disseminating public healthcare data and information	6.16 ± 1.73
7.	Able to use multiple methods when conveying healthcare and service information	6.24 ± 1.77
8.	Able to apply the relevant behavioral theories when designing information content	5.77 ± 1.91

Note: The descriptions of the items in the table are shortened versions and may differ from those in the original questionnaire.

**Table 3:** Analysis of inter-group differences and correlations regarding familiarity with, attitudes toward, and confidence in implementing HL practices.

Item	Familiarity			Attitude			Confidence		
	Mean	SD	F/t/R	Mean	SD	F/t/R	Mean	SD	F/t/R
Educational qualifications									
College for professional training	4.39	1.52	2.55	7.35	2.11	0.18	6.14	1.80	2.01
College	4.17	1.93		7.43	1.80		5.95	1.53	
Research institute (master’s degree)	5.50	1.58		7.75	1.47		6.96	1.23	
Presence of supervisory duties									
Yes	5.14	1.46	−1.65	7.89	1.86	−0.94	6.77	1.62	−1.67
No	4.24	1.88		7.38	1.50		6.00	1.02	
Region						0.53			0.90
Northern	4.19	1.96	0.54	7.33	1.82		5.90	1.73	
Central	3.98	1.92		7.10	1.74		6.09	1.14	
Southern	4.66	1.62		7.77	1.64		6.49	1.54	
Eastern & outlying islands	4.40	1.95		7.41	2.065		5.91	1.61	
HL education and training									
No	3.46	1.96	−3.29 *	7.73	1.90	1.32	5.92	1.71	−0.96
Yes	4.84	1.60		7.25	1.75		6.22	1.49	
Length of work experience			0.02			−0.05			0.00
Age			0.10			−0.10			0.05

Note: * *p* < 0.01.

**Table 4:** Analysis of the factors affecting CHPs’ level of confidence in implementing HL practices.

Predictive Variables	Estimated Value of B	Standard Error	Standardized Coefficient (β)	*t*	*p*	R^2^	F
Constant	0.97	0.50		1.95	0.05	0.57	58.96 *
Familiarity	0.44	0.06	0.51	7.28	<0.001		
Attitude	0.43	0.06	0.50	7.19	<0.001		

Note: * *p* < 0.001.

## Data Availability

No new data were created or analyzed in this review. Data sharing is not applicable to this article.
